# The Bacterial Enzyme RfxCas13d Is Less Neurotoxic Than PspCas13b and Could Be a Promising RNA Editing and Interference Tool in the Nervous System

**DOI:** 10.3390/brainsci11081054

**Published:** 2021-08-09

**Authors:** Qin-Wei Wu, Josef P. Kapfhammer

**Affiliations:** Department of Biomedicine, Institute of Anatomy, University of Basel, 4056 Basel, Switzerland; Qinwei.wu@unibas.ch

**Keywords:** neurotoxic, CRISPR, RfxCas13d, PspCas13b, Purkinje cells

## Abstract

RNA therapies using RNA editing and interference are currently being developed for neurological diseases. The CRISPR-Cas13 system, based on bacterial enzymes, holds great promise for developing efficient tools for RNA therapies. However, neurotoxic activity has been reported for Cas13a, and recent studies have reported toxic effects of PspCas13b and RfxCas13d during zebrafish and Drosophila embryonic development. It is important to investigate the safety of these bacterial enzymes in the context of the nervous system and neuronal development. In this study, we used mouse cerebellar Purkinje cells as a complex neuron type to test for the potential neurotoxic actions of RfxCas13d and PspCas13b. We found that PspCas13b significantly impeded the dendritic development of cultured Purkinje cells, similar to the neurotoxic action of Cas13a. In contrast, RfxCas13d did not exhibit a significant inhibition of dendritic development. A similar trend was found for axonal outgrowth. These results suggest varying neurotoxic properties for different Cas13 ortholog enzymes. We call for more studies to investigate, and possibly mitigate, the neurotoxicity of Cas13 proteins in order to improve the safety of the CRISPR-Cas13 system for RNA therapies.

## 1. Introduction

Ribonucleic acid (RNA) therapies have been successfully used to treat several neurological diseases, paving the way for the next-generation RNA therapies that are now in clinical development. Similar to antisense oligonucleotide therapies, CRISPR-Cas13-based RNA editing technologies offer a novel way to manipulate RNA information without altering DNA [1,2,3,4]. While most of the initial studies on gene-knockdown using Cas13 were done with Cas13a, more recently Cas13d and Cas13b were identified as related Cas13 enzymes of the Cas13 protein family in bacteria. These enzymes contain two higher eukaryote and prokaryote nucleotide-binding domains associated with ribonucleases [5,6,7,8,9,10,11]. Expression of Cas13d, together with guide RNA, can achieve knockdown of endogenous transcripts with high efficiency and specificity. Catalytically inactive Cas13d can also be used to manipulate alternative splicing, making it a promising tool for future therapeutic development [9]. Similar to the CRISPR-Cas13d variant, its ortholog Cas13b has great potential for use in a Cas13-based knockdown, as well as for editing transcripts containing pathogenic mutations by using catalytically inactive Cas13b [5]. The Cas13b-based toolkit has been further developed for the treatment of genetic diseases as it is able to recode RNA information [7]. Studies have explored the use of RNA knockdown and editing by CRISPR-Cas13 enzymes in the field of therapeutic agents for neurological diseases [3,4]. If this system was to be developed as a tool for therapeutic purposes, it is a prerequisite that no cellular toxic effects occur. We have shown previously that Cas13a has a distinct neurotoxic effect on developing nerve cells [6,12,13]. The Cas13d variant from *Ruminococcus flavefaciens XPD3002* (RfxCas13d) was shown to be an efficient tool when performing gene knockdown; lacking cellular toxicity not only in zebrafish embryos, but medaka, killifish, and mouse embryos [10]. However, recently, some cellular toxicity of Cas13d in flies was reported [11]. Similarly, a recent study found that the Cas13b proteins from *Prevotella sp. P5-125* (PspCas13b) and from *Porphyromonas gulae* (PguCas13b) did cause cellular toxicity during the embryonic development of zebrafish [10]. In view of the neurotoxic effect of Cas13a and the findings of cellular toxicity of Cas13d in flies [11] and Cas13b in zebrafish embryos [10], it is important to study the potential neurotoxic effects of Cas13b and Cas13d on developing neurons. In view of the rapid development of RNA editing technology as a potential treatment method for neurological diseases [1,2,3,4], more attention needs to be paid to potential safety concerns. In this study, we used developing mouse cerebellar Purkinje cells as a complex neuron type and expressed the bacterial proteins RfxCas13d and PspCas13b in mouse cerebellar Purkinje cells in culture. We then checked for possible neurotoxic effects of Cas13 enzymes, similarly to our previous study with Cas13a [13]. Our results demonstrate that RfxCas13d expression did not cause a significant reduction in Purkinje cell dendritic development, but that PspCas13b protein had a marked neurotoxic effect on the development of cultured Purkinje neurons with a significant inhibition of dendritic growth. The effect of RfxCas13d on Purkinje cell dendritic development is absent or much less strong compared to that of PspCas13b. Compared to other Cas13 proteins, RfxCas13d could thus be a promising candidate to explore RNA therapeutic tools for use in the nervous system.

## 2. Results

### 2.1. Purkinje Cell-Specific CRISPR-RfxCas13d Expression in Cerebellar Purkinje Neuron Cultures

**Figure 1:**
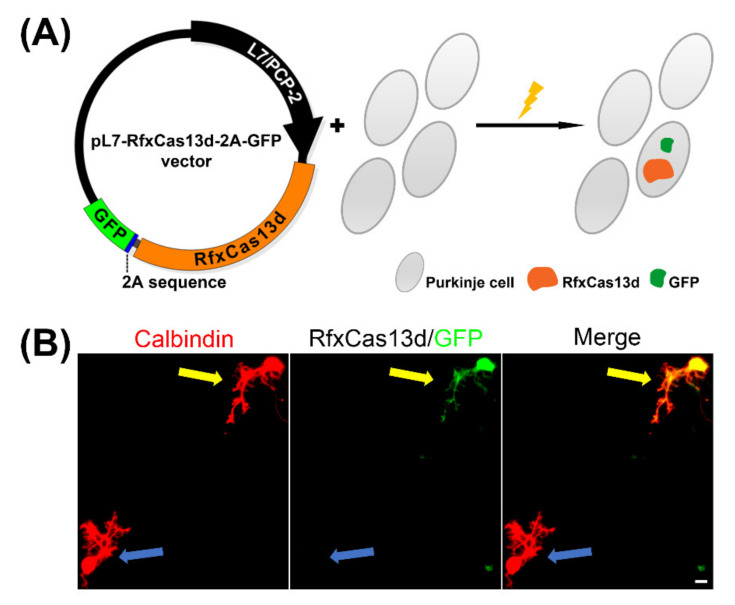
Purkinje-specific RfxCas13d expression. (**A**) A schematic view of the Purkinje-specific RfxCas13d expression construct; (**B**) Two Purkinje cells identified by the marker Calbindin (red) after two weeks in primary cerebellar cell culture. A RfxCas13d-2A-GFP transfected Purkinje cell (yellow arrow) and a non-transfected Purkinje cell (blue arrow) can be observed and distinguished by the presence of the GFP tagging protein. Scale bar is 20 μm.

In order to achieve sustained Purkinje cell-specific RfxCas13d expression, the pXR001: EF1a-CasRx-2A-EGFP vector (Plasmid #109049) was obtained from Addgene (Cambridge, MA, USA) as described in the STAR METHODS of the original study [9,10]. The RfxCas13d sequence was cloned from plasmid #109049 and was inserted into an expression vector with a L7/PCP2 Purkinje cell specific promoter to design pL7-RfxCas13d-2A-GFP (Figure 1A).

The expression plasmids for RfxCas13d, under the control of the L7/PCP2 promoter, were transfected into dissociated Purkinje cells on the day of culture setup. We successfully achieved Purkinje cell specific RfxCas13d expression of the indicated insert, which lasted for at least two weeks, by using the L7/PCP2 promoter [14]. pL7-RfxCas13d-2A-GFP was specifically expressed in the Purkinje cells which were identified by anti-Calbindin immunostaining; this is a neuronal maker of Purkinje cells. The use of a 2A sequence in the plasmid allows simultaneous expression of both the RfxCas13d and GFP [9,15]. In the culture wells, we could identify Purkinje cells with the expression of RfxCas13d-2A-GFP by staining with anti-GFP. This was in contrast to non-transfected Purkinje cells which were GFP-negative (Figure 1B).

### 2.2. CRISPR-PspCas13b, But Not CRISPR-RfxCas13d, Impedes Development of Cultured Purkinje Neurons

**Figure 2:**
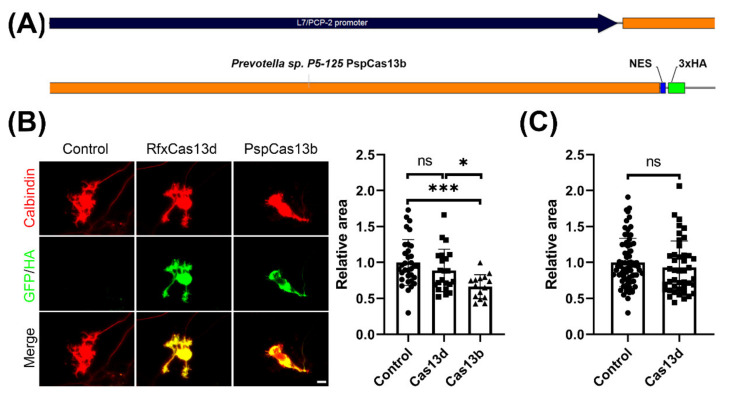
Purkinje-specific PspCas13b expression inhibits Purkinje cell dendritic growth. (**A**) A schematic view of the Purkinje cell specific PspCas13b expression vector. (**B**) The mean values of the Purkinje cell dendritic area were measured in three independent culture experiments. Representative images of transfected Purkinje cells are shown left. Scale bar is 20 μm. Control cells were non-transfected Purkinje cells from the same culture wells. Control: 1.00 ± 0.320, *n* = 33 cells; RfxCas13d 0.89 ± 0.295, *n* = 22 cells; PspCas13b 0.66 ± 0.166, *n* = 15 cells; Control vs. RfxCas13d, not statistically significant (ns); Control vs. PspCas13b, *p* = 0.0002 (***); RfxCas13d vs. PspCas13b, *p* = 0.0241 (*) in one-way ANOVA with Kruskal–Wallis test; Data are expressed as mean ± SD. (**C**) In order to exclude a negative effect of RfxCas13d, we increased the number of analyzed cells. The results confirm that RfxCas13d had no or only a minor negative effect on the Purkinje cell dendritic area. Control 1.000 ± 0.3365, *n* = 71 cells, RfxCas13d 0.929 ± 0.3707, *n* = 43 cells, difference was not statistically significant in two-tailed Mann–Whitney test.

We wanted to evaluate the potential toxic effects of Cas13b and Cas13d on cerebellar Purkinje cells, and thus we analyzed the dendritic morphology of Purkinje cells transfected with the respective constructs. The Cas13b vector pC0046-EF1a-PspCas13b-NES-HIV (Plasmid #103862) was obtained from Addgene (Cambridge, MA, USA) in order to design a Purkinje-specific CRISPR-Cas13b expression construct. In the original studies of PspCas13b enzyme, a PspCas13b-NES-HIV vector was used. NES-HIV refers to the fact that the C-terminal of PspCas13b was fused with the HIV Rev gene NES, which is a nuclear export signal [5]. As there are also 3xHA tagging sequences after the NES sequence in the original plasmid, we cloned the original engineered PspCas13b-NES-HIV sequence from the Addgene Plasmid #103862 into the pL7 vector, in order to make an engineered pL7-PspCas13b-NES-3xHA (Figure 2A). With this vector, we achieved Purkinje cell-specific expression of the pL7-PspCas13b-NES-3xHA.

As GFP expression by the pL7 vector was shown to have no effect on Purkinje cell dendritic development compared with non-transfected control Purkinje cells in previous studies [16], the dendritic area of Purkinje cells from pL7-RfxCas13d-2A-GFP and pL7-PspCas13b-NES-3xHA was compared to the dendritic area of non-transfected control Purkinje cells kept in the same electroporation and culture conditions. We found that transfection with the PspCas13b vector resulted in a marked reduction in the dendritic area, similar to the neurotoxic effect of LwaCas13a reported previously [13]. The mean for the dendritic area of PspCas13b-expressing Purkinje cells identified by anti-HA was significantly reduced to 66% of that of the control Purkinje cells, demonstrating a significant inhibition of dendritic growth by the PspCas13b protein (Figure 2B). In contrast, expression of RfxCas13d only caused a small reduction in dendritic development of Purkinje cells, which did not reach statistical significance (Figure 2B). Because the number of evaluated cells in these experiments was limited, we increased the number of cells analyzed. These experiments confirmed that RfxCas13d did not significantly affect Purkinje cell dendritic development (Figure 2B). Our findings show that RfxCas13d has no or only small neurotoxic effects on developing dendrites of cerebellar Purkinje cells, in contrast to PspCas13b or LwaCas13a [13].

We also looked at axonal outgrowth from the Purkinje cells (data not shown). As axons overlap extensively, it was difficult to quantify axonal outgrowth in these cultures with a mixture of transfected vs. non-transfected cells. We selected Purkinje cells that had little overlap with neighboring cells in order to quantify axonal outgrowth. The mean axonal length of the control group was set to one, and the length of the experimental groups were expressed as ratios of the control value (Control, 1.000 ± 0.2999, *n* = 15; Cas13d, 0.974 ± 0.2968, *n* = 11; Cas13b, 0.781 ± 0.3638, *n* = 10; Control vs. Cas13d, *p* = 0.7675; Control vs. Cas13b, *p* = 0.1495 in one-way ANOVA with Kruskal–Wallis test). These preliminary results confirm that the development and process outgrowth of Purkinje cells transfected with Cas13b is reduced, while transfection with Cas13d had no or only a mild negative effect.

## 3. Discussion

In this study, we evaluated the possible neurotoxic actions of two Cas13 endonucleases by expressing the CRISPR-RfxCas13d and PspCas13b proteins in Purkinje cells and analyzing their dendritic development. We found that Cas13b had a pronounced neurotoxic effect, similar to that previously shown for LwaCas13a [13]. In contrast, Cas13d had no significant neurotoxic effect on Purkinje cell dendritic development. Our results show distinct differences in the neurotoxic properties of Cas13 ortholog enzymes. As transfection of RfxCas13d resulted in no significant inhibition of Purkinje cell dendritic development and axonal outgrowth, it is a promising candidate for the use of the CRISPR-Cas13 system in neuronal cells.

In two recent studies from our laboratory, the potential toxic effects of LwaCas13a from *Leptotrichia wadei* have been demonstrated during neuronal development [12,13]. The inhibition of Purkinje cell dendritic development by the LwaCas13a is not due to the classical collateral activity of Cas13 enzymes, but instead is a property of the Cas13a protein itself [6,13]. Our current findings on Cas13d and Cas13b also show that Cas13b has a strong negative impact on Purkinje cell dendritic development, while Cas13d was not obviously toxic for neuronal development. These findings corroborate recent studies suggesting that potential toxic effects of Cas13 may occur, in particular, during developmental stages. In a recent report, toxic effects of Cas13 variants in zebrafish embryos have been investigated [10]. In these studies, Cas13d did not inflict toxicity upon zebrafish embryos, but a high percentage of abnormal morphologies among zebrafish embryos was induced by expression of Cas13b, suggesting that Cas13b, but not Cas13d, is toxic in zebrafish development [10].

Our finding that RfxCas13d (in contrast to Cas13a and Cas13b) did not show a significant effect on Purkinje cell dendritic development and axonal outgrowth establishes it as a promising candidate for exploring CRISPR-Cas13-based tools for RNA editing and interference, which may even be used during development and for treatment strategies in human diseases. Nevertheless, for several reasons, the use of RfxCas13d for RNA interference should only be considered with caution. First, in this study, we only observed the morphology of Purkinje cell dendritic development after RfxCas13d expression in dissociated cerebellar cultures. More complicated phenotypes and effects, such as axon development and guidance or synapse formation, need to be studied and explored under in vivo physiological conditions in transgenic mouse models. Second, Kushawah et al. found that zebrafish embryos injected with 400 pg and 600 pg of mRNA encoding for RfxCas13d had an increased percentage of deformed zebrafish—up to 30% (supplementary information in their study) [10]—indicating that with higher amounts of Cas13d present a negative effect might become relevant. Third, RfxCas13d was reported to cause negative effects in developing Drosophila larvae [11].

The US Food and Drug Administration has recently approved the first RNA interference therapy and this will probably open a path for future RNA therapies in human patients. Some companies have tried to seek approval for Cas13-based RNA editing technologies in the clinical treatment of genetic diseases, according to news reports [2]. Therefore, our findings about the neurotoxic effects of Cas13d and Cas13b in developing neural cells are a reminder to pay more attention to, investigate, and understand the mechanisms of the toxic properties of Cas13 enzymes. Only when the relative toxic mechanisms are known and understood will it be possible to design mitigation strategies that make future clinical use of this technology possible, through robust protection against any dangers and potential risks. Further studies to better define the toxic properties of the different Cas13 proteins will be required before RNA therapies for future clinical use can be developed and safely applied.

## 4. Materials and Methods

### 4.1. Mice

All experiments were carried out in accordance with the EU Directive 2010/63/EU for the care and use of laboratory animals, were approved by the veterinary office of the canton of Basel, and were permitted by Swiss authorities. FVB mice were used for primary mouse cerebellar cell cultures.

### 4.2. Plasmid Construction

Engineered RfxCas13d and PspCas13b coding sequences were cloned into pL7 vectors for cell-specific expression in Purkinje cells [14] and prepared using the EndoFree Plasmid Maxi Kit (QIAGEN) according to the manufacturer’s protocol. The engineered RfxCas13d sequence was PCR amplified from the pXR001: EF1a-CasRx-2A-EGFP vector (Addgene plasmid #109049). The engineered PspCas13b sequence was PCR amplified from the PspCas13b vector pC0046-EF1a-PspCas13b-NES-HIV (Addgene plasmid #103862), a gift from Feng Zhang.

### 4.3. Cultures of Cerebellar Purkinje Cells and Electroporation Procedure

Primary mouse cerebellar Purkinje cell cultures were prepared from neonatal mice according to the previously described protocol [16]. Briefly, cerebella from postnatal day 0 mice were dissected, dissociated, and plated on glass chambers coated with Poly-D-lysine. Indicated vectors were introduced into Purkinje cells by transfection with a Neon Transfection System (Thermo Fisher Scientific, Massachusetts, United States) using the following settings: Pulse voltage 1200 V, Pulse width 30 ms, Pulse number 1. Cells were incubated in incubation medium (90% Dulbecco’s modified Eagle medium/F-12 nutrient-based medium, 1% N2 supplement, 1% glutamax, and 10% FBS). 2–4 h after transfection, 500 μL DFM supplemented with 1% N2 and 1% glutamax was added to each well. After that, half of the medium was refreshed every 4 days. The media and supplements were from Life Technologies, Zug, Switzerland. Cells were kept in culture for 2 weeks before fixation.

### 4.4. Immunocytochemistry

Primary mouse cerebellar Purkinje cells were fixed in 4% paraformaldehyde for 30 min at room temperature. All reagents were diluted in 100 mM phosphate buffer (PB), pH 7.3. Fixed cells were incubated with primary antibody diluted in blocking solution (PB + 3% non-immune goat serum + 0.5% TritonX-100) for 1 h at room temperature. After washing with PB, the corresponding fluorescence-conjugated secondary antibodies were added to the cells in PB containing 0.1% Triton X-100 for 2 h at room temperature. The following primary antibodies were used: rabbit anti-Calbindin D-28K (1:500, Swant, Marly, Switzerland); mouse anti-Calbindin D-28K (1:500, Swant, Marly, Switzerland); Guinea pig anti-Calbindin (1:4000, SYSY, Göttingen, Germany); rabbit anti-GFP (1:2000, Novus, Zug, Switzerland); chicken anti-GFP (1:2000, Abcam, Cambridge, United Kingdom); and mouse anti-HA tag (1:1000, Invitrogen, Massachusetts, United States). The staining was visualized with anti-mouse Alexa 568, anti-guinea pig Alexa 568, anti-chicken Alexa Fluor Plus 488, and anti-rabbit Alexa 488 secondary antibodies (1:2000, Molecular Probes, Eugene, OR, USA). The stained cells were mounted with Mowiol (Sigma-Aldrich, Buchs, Switzerland). The images were captured on an Olympus AX-70 fluorescence microscope equipped with a Spot Insight digital camera.

### 4.5. Quantitative Analysis

The quantification of dendritic area was performed by an image analysis program described previously [16]. Briefly, the average value of control Purkinje cells was set as 1. Non-transfected Purkinje cells located close to the transfected Purkinje cells, identified by tagging protein expression, and grown in the same transfection and culture conditions were taken as a control in order to ensure a comparable growth environment. An image analysis program (ImageJ) was used to trace the outline of the Purkinje cells yielding the area covered by the cell body and dendritic tree. Only Purkinje cells which had little overlap with neighboring cells were selected for the quantification of axonal outgrowth. ImageJ was used to measure the length of the Purkinje cell axons. The average axonal length of the control group was set as 1, and the length of the experimental groups were expressed as ratios of the control value. The data were analyzed using GraphPad Prism software (San Diego, CA, USA). The shown images were linearly adjusted in brightness and contrast. The statistical significance of differences in parameters was assessed by a non-parametric Kruskal–Wallis test or a two-tailed Mann–Whitney test. Confidence intervals were 95% and statistical significance was assumed with *p* < 0.05.

## Data Availability

The original data are available upon request from the authors. Because of the strong overlap of the axons and the mixture of transfected and non-transfected cells in each individual culture, we could only quantify axonal outgrowth from Purkinje cells for which there was only little overlap with axons from adjacent Purkinje cells.
